# Message framing, non-conscious perception and effectiveness in non-profit advertising. Contribution by neuromarketing research

**DOI:** 10.1007/s12208-021-00289-0

**Published:** 2021-06-02

**Authors:** Ana C. Martinez-Levy, Dario Rossi, Giulia Cartocci, Marco Mancini, Gianluca Di Flumeri, Arianna Trettel, Fabio Babiloni, Patrizia Cherubino

**Affiliations:** 1 Department of Communication and Social Research, Sapienza University of Rome, Rome, Italy; 2 Department of Business and Management of LUISS Guido Carli, Rome, Italy; 3 Department of Molecular Medicine, BrainSigns Srl, Sapienza University of Rome, Rome, Italy; 4 BrainSigns Srl, Rome, Italy; 5 Department of Molecular Medicine, Department of Computer Science, BrainSigns Srl, Sapienza University of Rome, Rome, Italy

**Keywords:** Non-Profit Advertising, Effectiveness, Emotion, Neuromarketing, Call to action

## Abstract

Advertising for non-profit organizations through television commercials is a valuable means of communication to raise awareness and receive donations. When it comes to social aspects, personal attitudes such as empathy are significant for reinforcing the intention to donate; and the study of eliciting emotions has critical attention in the literature, especially some types of emotion, such as guilt which mediates empathy. Different methodologies have been used to measure consumer emotions when faced with TV ads stimuli: mainly traditional techniques such as interviews or questionnaires after the ads viewing. In the last ten years, there has also been a great interest in new neuroscience techniques applied to measure emotional and cognitive reactions by physiological signals, frame by frame. Our research has applied neuromarketing technologies during the observation of a UNHCR commercial promoting legacy calls. The objective was to study cognitive and emotional reactions in order to increase the effectiveness whilst having the possibility to verify the results by measuring the benefits in terms of calls from contributors. The purpose of this research is to empirically prove the impact in calls thanks to changes in the message framing strategy in non-profit advertising suggested and measured by neuromarketing techniques. Particularly we measured the cerebral activity through an electroencephalogram to obtain an Approach-Withdrawal Index (AW); the heart rate and galvanic skin response through different sensors in the palm of one hand, to obtain an Emotional Index (EI), and finally, eye fixations through an eye tracker device to obtain the visual attention on key visual areas of the ads. After these indicators’ recordings on a sample of subjects, some suggestions to modify the advertising were made to create a more effective campaign. The results compared, those elicited by the first version of the spot (LVE) and those by the second version (HVE), confirmed that (1) the number of sellable and legacy calls increased with the message framing strategy modified in the second spot (HVE), (2) a lower cognitive and emotional reactions have been obtained in the final section of HVE, (3) the visual attention on the key information of the phone number to call, in the final call to action frames(CTA), was higher in HVE than in the first version of the spot (LVE), (4) the cognitive approach increased during the same CTA frames in HVE.

## Introduction

How to improve creativity effectiveness has become a central issue in the advertising sector in continuous multimedia communication. More particularly, strategies for non-profit advertising organizations have received much attention in recent years(Mirzaei et al., 2020; Nguyen & Faulkner, 2020; Olofsson & Funke, 2020). These organizations oversee helping people in need, and each one has a mission and vision focused on a particular objective. Depending on the mission, different strategies are required to create efficient creativity. The most studied measure of effectiveness in this sector within marketing research is the declared intention to donate (Dos Santos et al., 2017).

Moreover, there is an active debate in the literature covered in this paper about using positive or negative images in non-profit advertising campaigns to motivate real actions for donations. In this context, empathy is an essential factor in inciting donation (Verhaert & Van den Poel, 2011). Most studies focus on studying emotions, considering that some emotions, such as guilt, mediate empathy.

How to generate a non-profit commercial effective? On the one hand, advertising with only positive images may cause a lack of guilt, while an advertisement with only negative images could create an absolute refusal from the message. On the other hand, a potential donor must clearly understand how to donate, then the call to action of non-profit advertising must attract the donors and capture their interest. The importance of publicity campaigns is always relevant. In this time of the Coronavirus disease (COVID-19) emergency more than ever as the economic situation in the most developed countries has deteriorated sharply due to the restrictive measures to avoid the pandemic's spread (McKibbin & Fernando, 2020). Due to the COVID-19 pandemic, the global health situation has added other causes to those already existing in the countries supported by non-profit organizations (Glass et al., 2020).

Previous work in the non-profit sector advertising has been limited to measure consumer responses with traditional marketing tools, such as interviews or questionnaires, without considering the consumers' non-conscious, implicit response (Kim, 2014). However, it has been confirmed that these measures to evaluate emotions may not be accurate, or worse, may give wrong results conditioned by factors external to the stimulus, such as the pressure to answer what the interviewer wants to hear or simply because people do not always know how to interpret in words the emotions they are feeling at any given moment (Lagast et al., 2017).

In response to the need to use implicit measurement methodologies, the past decade has renewed importance in using neuroscience tools to study consumer behaviour. Neuromarketing has often been applied with its techniques to investigate consumer insights(Cartocci, Cherubino, et al., 2019), demonstrating that it helps to integrate the knowledge available in the existing literature on the role of emotions also for non-profit advertising organizations (Martinez-levy et al., 2017). Despite this interest, no one, to the best of our knowledge, has done an empirical study to improve the efficacy of a real non-profit advertising campaign by neuromarketing techniques with successful results confirmed by real data of the benefits generated. The present paper mainly aims to empirically validate literature findings regarding the theories about the role of emotion in non-profit advertising by applying neuromarketing techniques and those theories in a real case study and measuring obtained benefits.

The paper is organized as follows. The first section gives a brief overview of the existing literature about the more discussed and advanced theory regarding the role of emotion for creating successful non-profit advertising and about neuromarketing techniques that can be used to check emotional and cognitive perception. In the second section, a performed test study is described with its specific neuromarketing methodology. The third section describes the results. Discussion and conclusions are drawn in the final section.

## Literature review

### Effectiveness of non-profit advertising

Non-profit advertising ranges from helping those in need, including poverty, slavery, sickness, to motivate those in need of protection, to more individualistic behavioural changes, such as safe driving or anti-smoking campaigns (Shanahan et al., 2012). Non-profit organizations have to engage the potential donors rising awareness to other people needs and motivating to help by donations. Then, non-profit advertising is considered adequate when there is a positive impact of a donation (Caviola et al., 2014). Advertisers must convince the individuals that their contribution is worthwhile and make a difference (Manrai & Gardner, 1992). There is a vast amount of literature about increasing donation intention (Cheung & Chan, 2000; Merchant et al., 2010; Ramanath, 2016; Ranganathan & Henley, 2008). However, there is still much to research in this field regarding the impact of advertising on real donations.

Marketing campaigns can be created to be more targeted and useful for generating donations by a better understanding of what drives and motivates people to donate to a charity (Kashif et al., 2015). Several research on donations in the non-profit sector found the personal empathy value significant (Basil et al., 2008; Bergh & Reinstein, 2020; Martinez-levy et al., 2017; Verhaert & Van den Poel, 2011). In this respect, the impact of empathy on charitable donation intention is fully mediated by guilt responses (Basil et al., 2008), and there is a considerable amount of literature on guilt to be another relating factor for an individual's intention to donate to charity (Basil et al., 2008; Brennan & Binney, 2010; Urbonavicius et al., 2019). Identifying individual victims is essential for empathy and willingness to favour those who are suffering (Bleiker et al., 2013). Although many shreds of evidence suggest that guilt appeals can be a useful tool for influencing donors behaviour (Bennett, 1998) and that emotion plays a crucial role in the prediction of effective non-profit advertising campaigns (Martinez-levy et al., 2017), further work needs to be done to establish how it happens and how it can be assessed. It is well known that the emotions evoked with advertising can be negative, neutral or positive (Vecchiato et al., 2014) and that emotional contagion is a mediator for the identifiable victim effect (Small & Verrochi, 2009).

Human beings' negative state induces helping behaviour through the drive to reduce their negative feelings through altruistic behaviour, such as donating to a charity (Merchant et al., 2010). Some discrepancies exist in the current literature of negative emotions and helping behaviour within the non-profit sector. While some authors supported the positive relationship between negative emotions, empathy and helping behaviours (Bagozzi & Moore, 1994), other authors found that too strong images of disgust, while invoking higher levels of empathy, showed lower donation intention (Allred & Amos, 2018). It seems that elicited negative emotions help to motivate donation but avoid too strong negative ones or abrupt interruption. When negative emotions are evoked, it results in greater sympathy for the victim and encourages prosocial behaviour. Numerous researchers attempted to find the most effective modes of charity advertising through the type of emotion, but most studies did not differentiate between money and time donations. Showing helped beneficiaries can attract more volunteering, whereas showing needy beneficiaries can attract more monetary donations (Kim, 2014). It underlines the critical importance to consider whether and how to show a helped beneficiary -eliciting positive emotions and volunteering-motivation; or a needy beneficiary—eliciting negative emotions and monetary donations- when creating non-profit advertising.

It seems evident that the impact of eliciting the right emotion/mix of positive and negative emotions with the right images is a crucial issue in a non-profit advertisement.

Regarding the method of helping, the call to action (CTA) of non-profit advertising has always to communicate information on "what to do" to donate with images, text and numbers (website, call numbers, bank account and similar). CTA is usually at the end of the spot when all other messages have been communicated. So, the risk of a lack of attention to this final part requires creativity strategies with images and sounds capable of activating the viewers' effective decoding and cognitive approach. Then it is vital to create message framing strategies that generate the right impact in the visual attention and an alive cognitive approach of viewers to CTA's elements.

So, it seems clear that we must differentiate between cognitive or emotional framing (Huang & DiStaso, 2020; Lim et al., 2018). A rational appeal relates to receivers' rationality by providing objective information, whereas an emotional appeal features more subjective, emotional expressions and other emotion- eliciting strategies (McKay-Nesbitt et al., 2011; Yoo & MacInnis, 2005). To enhance a non-profit campaign's effectiveness, it is crucial to have an appropriate message framing the spectator to feel involved in both appeals.

### Neuromarketing research: emotional / cognitive engagement and visual attention measures

The last decade has seen a massive growth in brain imaging techniques and biometrics to analyze consumer responses to commercial stimuli to assess the emotional and cognitive process through which these operate (Hsu & Chen, 2020; Lim, 2018a, 2018b; Spence, 2019). The traditional techniques measure cognitive and emotional experiences only as verbally expressed at the conscious level. Instead, it is possible to distinguish the unconscious states related to processes that play a crucial role in influencing behaviours by using brain imaging techniques, integrating what can be found by verbal or written self-reports (Cherubino et al., 2019). Neuroscientists started to investigate the brain activity gathered during TV commercials by measuring variables linked to cognitive and emotional engagement (Vecchiato et al., 2014). Those developments have led to a relatively new discipline called neuromarketing. It seeks to investigate different brain areas while experiencing marketing stimuli to find and report the relationship between customer behaviour and the neurophysiological system (Cherubino et al., 2019). Several studies have been conducted to evaluate the efficacy of commercial advertising. Ioannides and colleagues have employed MEG to study the neuronal responses in subjects viewing the same TV advertisements (Ioannides et al., 2000) as used by Ambler and Burne (1999). The results show that cognitive advertisements rather than affective ones activate cortical centres associated with the executive control of working memory and maintenance of higher-order representations of complex visual material. Interestingly, neuronal responses to an affective visual material seem to exhibit more significant intersubject variability than responses to a cognitive material. Young has used the EEG to detect putative "branding moments" within TV commercials (Young, 2002). Other neuromarketing studies have been conducted for the assessment of the efficacy of TV advertising stimuli (Astolfi et al., 2008, 2009; Cherubino et al., 2016; Dimpfel, 2015; Ohme et al., 2009, 2010; Vecchiato et al., 2010, 2011, 2014; Vecchiato et al., 2013), to investigate the consumer's gender differences during the observation of TV commercials (Cartocci et al., 2019; Martinez-levy et al., 2017) and for social campaigns (Cartocci et al., 2018; Cartocci et al., 2019; Martinez-levy et al., 2017; Modica et al., 2018; Orzan et al., 2012).

In 2008, Hubert and Kenning reported more than 800,000 Google hits for the term 'Neuromarketing' (Hubert & Kenning, 2008). In 2018, the same search yielded over 3 million hits underlining the rising interest in this topic, and there was an evolution of academic interest in neuromarketing*.* Nowadays, the total number of papers published with the keyword 'Neuromarketing' is approximately 24,000 (source: Google Scholar in January 2021). Such interest is justified by the possibility of investigating the unconscious responses to the proposed commercial stimuli to derive conclusions about the adequacy of such stimuli in terms of emotional and cognitive engagement for the consumer. There are a few fundamental dimensions that organize an emotional and cognitive response. The most assumed dimensions are valence, arousal and motivation(Davidson, 1999; Russell & Barrett, 1999).

On the one hand, the motivation can be obtained by measuring variations of the prefrontal and frontal cortex (PFC and FC, respectively) (Davidson, 1999). Its role in emotion is well recognized (Davidson, 2002). Electroencephalographic (EEG) spectral power analyses indicate that the anterior cerebral hemispheres are differentially lateralized for approach and withdrawal motivational tendencies and emotions. Precisely, frontal EEG asymmetry primarily reflects levels of approach motivation (left hemisphere) versus avoidance motivation (right hemisphere) (Vecchiato et al., 2014). So, it is possible to link some properties of the collected EEG rhythms during the vision of some TV advertisements with the consumers' overt preferences.

On the other hand, relevant studies often point to relationships among valence, arousal, and autonomic nervous system responses (Bradley & Lang, 2000; Kop et al., 2011; Posner et al., 2005). The galvanic skin response (GSR) for arousal and the heart rate (HR) for valence are the physiological variables often used to describe variations of emotional states (Russell, 1980). The HR and GSR are usually measured simultaneously with other brain tools (i.e., EEG).

Neuromarketing techniques include other devices such as Eye Tracker (ET) to measure the impact of TV advertising. It is used to obtain information about where visual attention is placed on certain advertising elements and how long each fixation lasts. Based on the relationship between visual attention and eye movements (Hoffman, 1998), the ET is a useful neuromarketing research tool. It records where and what the person is looking at (eye fixations), the time of fixations spent on a specific Area Of Interest (AOI), the movement of the eyes concerning the subject' head to get information about specific patterns of visualization, pupil dilation, and the number of blinks (Veneri et al., 2010; Zurawicki, 2010). The advertising sector has benefited the most from this technique (Dimpfel, 2015; Rossi et al., 2017). Obtaining measurable neurophysiological parameters, collected through direct analysis of the measured cognitive and physiological, emotional response and visual attention in response to non-profit advertising observation, represent an area of exciting questions and vital opportunities.

Despite the interest above, no one, to the best of our knowledge, has already done an empirical study applied to a real non-profit advertising campaign with neuromarketing techniques collecting real data about benefits. For this study, we obtained exciting cues derived from such techniques which were impossible to assess differently. These cues considered with the light of the theory about no-profit promotion indicated opportune changes in a non-profit spot's creative strategy: 1) elicit not only positive but also negative emotion and emphasize the beneficiary in need, 2) avoid confusion in the CTA plot in the final part in order to maximize attention and lively cognitive approach. Some cues, evidenced by neuromarketing indexes, like no negative emotion or low eye fixation on CTA information, have been translated into indications of changes in the framing message strategy of the non-profit spot coherent with the theory, whose effectiveness has been confirmed in terms of real calls and donations (in-kind provided by the non-profit organization).

Accordingly, before performing the study, we expected that:

-
  - H1: Right changes in the message framing communication can successfully induce an increase in effectiveness measurable in data about the benefits.
-
  - H2: Changes in the message framing of the spot showing without too much modesty war images with more and identifiable victims can elicit adequate emotion modulation (i.e. including also negative/guilty emotion)
-
  - H3: Changes in the message framing communication of the final CTA in the spot to avoid confusion and distraction can elicit greater visual attention to the phone number for information and a higher cognitive approach.

## Material and methods

This study aimed to evaluate the neurophysiological responses to two spots provided by the Italian UNHCR association in charge of helping refugees. The first spot^1^ provided by the UNHCR association was a non-profit spot already aired in Italy (prior to October 2017) but failed to elicit an increase in calls for donation in return; the second spot^2^ was a modified version of the first one as suggested by non-profit communication theory and by neuromarketing methods, intending to improve the performances of the first spot once aired. In light of each spot's content, LVE will refer to the spot in the test session one as "low victim effect" because the images showed mainly the UNHCR intervention (i.e. helping war victims) without using clear images of war victims. Instead, HVE will refer to the spot in test session two as "high victim effect" because more images of the victims were shown.

### Participants

The study took place in Italy and involved 72 healthy volunteers (50% Male, M_age_ = 37.53 ± 10.87), sampled from Rome and Milan, two main Italian cities: half of the population was recorded in Rome another half was recorded in Milan. Informed consent was obtained from each participant after explaining the study approved by the local institutional ethics committee. The experiment was conducted following the principles outlined in the Declaration of Helsinki of 1975, as revised in 2014 (World Medical Association Declaration of Helsinki (2014)).

### Stimuli

The study was divided into two experimental recording sessions. The first session aimed to evaluate the neurophysiological responses toward LVE (60s) aired in Italy provided by the UNHCR association that failed to improve KPIs such as return in calls and donations favouring refugees (data not shown, evaluated by UNHCR itself). In the second session HVE was evaluated according to suggestions from the literature that indicate an effective campaign (Peters, 2019) and from the neurophysiological evidence highlighted in the first session of the study (poor emotional engagement during the whole spot and low approach in the final part of the spot with the CTA information). Taking this information into account, two parts of the spot were identified as worthy of attention; thus, the spot was modified as follows: 1) inserted more explicit images of war and war's victims (at sec. 27) in order to elicit punctual changes in emotion and evoke empathy in the viewers and 2) use of visual effects in the final CTA with call number for information more evident and synchronized with voice over to better attract the viewer.

The spots were presented randomly in an ad-hoc advertising break with six other spots as distractors. In order to have more familiar fruition of the stimuli and, thus engage both participants chronic disposition and situational priming concerning information processing, the final video presented to the participants was composed by a selection of 60s documentary (chronic disposition)-a short clip extracted from an Italian translation of "Earth" documentary from BBC- with the advertising break (situational priming) at the end of the clip (Lim, 2015). The documentary part of the video was used as a baseline.

### Procedure

A between-subjects experimental design (LVE vs HVE) was used. 36 subjects were shown LVE spot in the first recording session, while the other 36 were shown HVE spot in the second recording session.

**Fig. 1:**
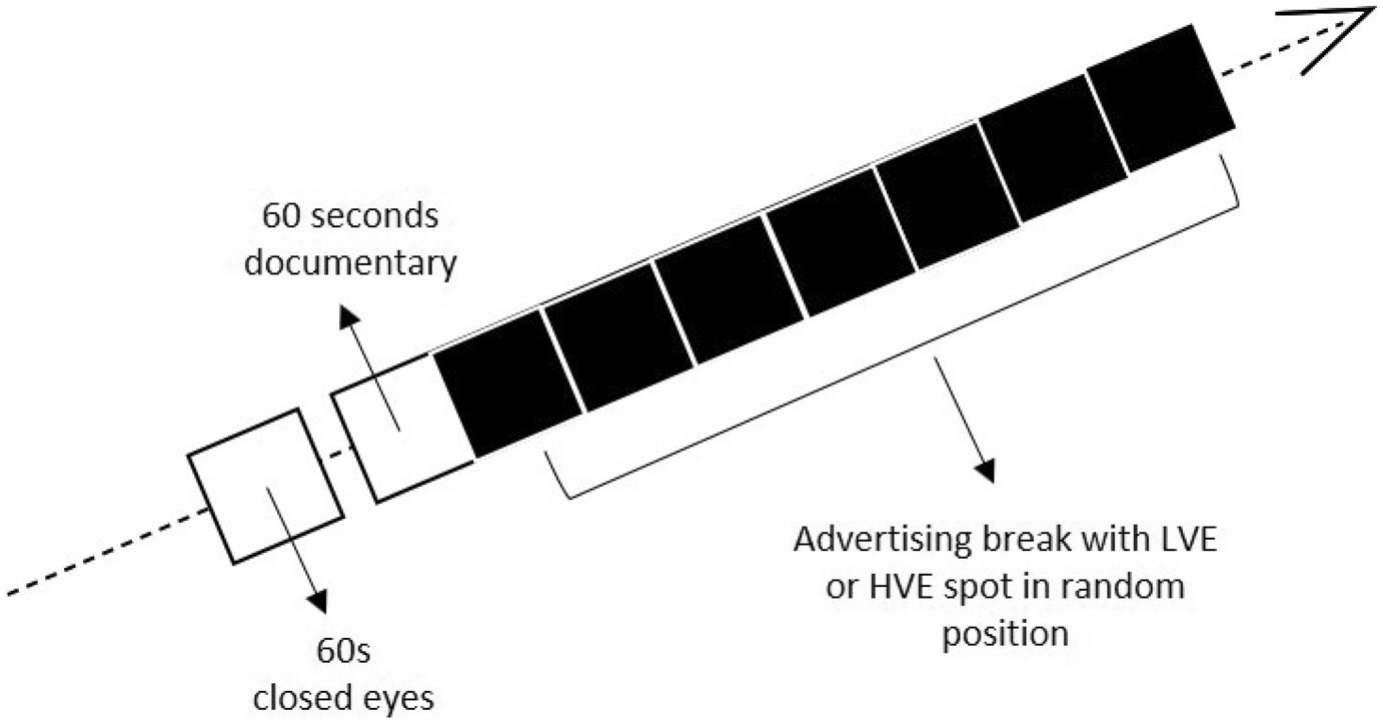
Experimental flow in both recording sessions

The participant was sitting in a comfortable chair in front of a 24" pc screen and was explained the experimental procedure. After signing the informed consent, an experimenter put the neuromarketing tools on the participant to acquire neurophysiological data throughout the video's whole vision. Before the video presentation, we recorded 60s while the participant was resting with closed eyes. Then the video was shown to the participants. Each participant session lasted about 30 min. Experimental flow can be seen in Fig. 1.

### Data acquisition and processing

#### EEG recording and cognitive approach-withdrawal index

The EEG activity was recorded using ten electrodes (Fpz, Fp1, Fp2, AFz, AF3, AF4, AF5, AF6, AF7, AF8) placed on the frontal portion of the scalp of participants using a portable 21-channels system (BEmicro, EBneuro, Italy). Although the system allowed up to 21 channels, a ready-made headband with ten electrodes placed over the prefrontal and frontal cortex was used since only this cortical area (prefrontal and frontal) was of interest in the present study and reduced the system's invasiveness and increased the comfort of the participant when compared with traditional EEG caps. The reference and the ground electrodes have been placed respectively on the left and right earlobes. The signals have been acquired at a sampling rate of 256 Hz, and the impedances were kept below 20kΩ. After the acquisition phase, the raw EEG signal was digitally pre-processed using the EEGLAB Matlab toolbox (Delorme & Makeig, 2004). Firstly, a notch filter (50 Hz) was applied to reject direct current interference. Secondly, the gathered signal has been band-pass filtered by a 5th order Butterworth filter ([2 ÷ 30] Hz) to reject the continuous component and high-frequencies interferences, such as muscular artefacts. Then, the Independent Component Analysis (ICA), in particular, the SOBI algorithm (Belouchrani et al., 1997), has been applied to EEG data in order to identify and remove the component related to eye-blinks and eye movements, since their contribution overlaps the EEG bands of interest (Di Flumeri et al., 2016). The component has been manually selected to be removed, and after that, the EEG signal has been reconstructed. Furthermore, to clean the EEG signal as much as possible, after these conservative steps (until now, no EEG data has been lost), the EEG signal segments still affected by artefacts have been automatically detected and rejected. To compute the activity of the cortical areas of interest in a specific frequency band, the Global Field Power (GFP) was then computed. This measurement summarises the synchronization level of brain activity over the scalp surface (Lehmann & Michel, 1990). GFP is computed from a specific set of electrodes by performing the sum of squared values of EEG potential at each electrode, averaged for the number of active electrodes, resulting in a time-varying waveform related to the increase or decrease of the global power in the analyzed EEG. The GFP formula is presented in the following:

$$GFP_{\vartheta,Frontal}=\frac1N\sum_{i=1}^Nx_{\vartheta_i}^2\left(t\right)$$

where θ is the considered EEG band on the frontal cortical area, N is the number of electrodes included in the area of interest, and *i* is the electrodes' index. The formula defining the frontal alpha asymmetry (Approach Withdrawal (AW)) index is as follows:

$$AW = GFP\alpha \_right - GFP\alpha \_left$$

where the GFPα_right and GFPα_left stand for the GFP calculated among right (Fp2, AF4, AF8, and AF6) and left (Fp1, AF3, AF5, and AF7) electrodes, respectively, in the alpha (α) band. Higher frontal alpha asymmetry values, reported by the participants, stood for an approach motivation toward the stimulus, while lower frontal alpha asymmetry values stood for a withdrawal motivation (Davidson, 2004). The AW Index value was estimated for each second and then standardized based on each experimental subject's baseline (documentary in the film).

#### Autonomic data recording and emotional index

The Blood Volume Pulse (BVP) and Galvanic Skin Response (GSR) were recorded with the Shimmer System (Shimmer Sensing, Ireland) with a sampling rate of 64 Hz. For the recording of these signals, two electrodes are placed on the palmar side of the middle phalanges of the second and third fingers on the non-dominant hand of the participant in order to acquire the GSR signal according to published procedures (Boucsein, 2012) and a photoplethysmography sensor is placed on the thumb of the same hand for the BVP recording. To obtain the heart rate (HR) signal from the BVP, it has been used the Pan-Tompkins algorithm (Pan & Tompkins, 1985).

The constant voltage method (0.5 V) is employed for the acquisition of the GSR, then, by using the LEDAlab software (Benedek & Kaernbach, 2010), the tonic component of the skin conductance (Skin Conductance Level, SCL) is estimated. To combine GSR and HR signals producing a monodimensional variable that returns the emotional state of subjects, the EI is defined by considering the GSR and HR signals (Vecchiato et al., 2014). We refer to the effects plane (Posner et al., 2005; Russell & Barrett, 1999), where the coordinates of a point in this space are defined by the HR (horizontal axis) and the GSR (vertical axis). Several studies have highlighted that these two autonomic parameters correlate with valence and arousal, respectively (Mauss & Robinson, 2009). The EI interpretation implies that the higher the value, the more emotional engagement experienced by the subject is, and vice versa. The GSR and HR values were estimated for each second and then standardized based on the baseline (documentary in the film). The EI has been calculated for each second (Vecchiato et al., 2014).

#### Eye-tracking recordings and visual attention

Eye-tracking data have been acquired by Tobii Pro X2-30 screen-based eye tracker with a sampling frequency of 30 Hz, in order to identify eye fixations on the proposed stimuli. As the first step, all the artifactual or not physiological point of gaze were automatically removed. Then, eye-tracking data were analyzed with Tobii studio 3.4.8 for the extraction of information about fixations in each area of interest (AOI), such as the number of fixations on each AOI; in this case, the AOI is related to the CTA elements presented during the spot. Based upon the total number of fixations recorded on the screen throughout the movie, the percentage of fixations eye-tracking metric has been performed for each subject to evaluate the visual attention elicited by each specific AOI. It has been obtained by dividing each participant's number of fixations for each AOI by the total number of fixations recorded on the screen throughout the movie for each participant.

## Results

### Real calls for the charity

The HVE spot was launched on-air on television after the changes in the creativity strategy suggested with the neuromarketing results in LVE. The UNHCR organization communicated a positive impact on real calls: + 553% in legacy calls (people that calls for getting more information about the testamentary legacy) that lead to a 237% increase in sellable calls (people that subscribe for the testamentary legacy) respect to the period before the HVE airing (all data provided by UNHCR association). This increase in calls confirms our H_1_.

### Trend of both spots by seconds

**Fig. 2:**
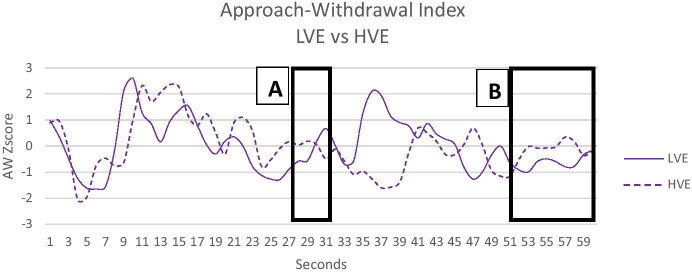
Approach-Withdrawal Index for LVE and HVE. Black boxes (**A** and **B**) represent the modified segments. The war images in **A** and the CTA in **B**

The IE and AW indexes trend change considerably after modifying the creativity with victim images in the war from the 27 s until the 30 s of the spot (See box A in Fig. 1 and Fig. 2). In particular, the AW becomes increasingly negative in the HVE, unlike the LVE. On the other hand, the EI is always at the same average positive level for LVE during the entire spot. Instead, for the HVE, it reverses after the victim's images modification becoming negative. This evidence highlights the impact of the new explicit images on creativity. It is important also to notice the change in the AW's trend during the final CTA with an increase in the cognitive approach (see box B in Fig. 1).

### Images of war victims effect in neurophysiological response

**Fig. 3:**
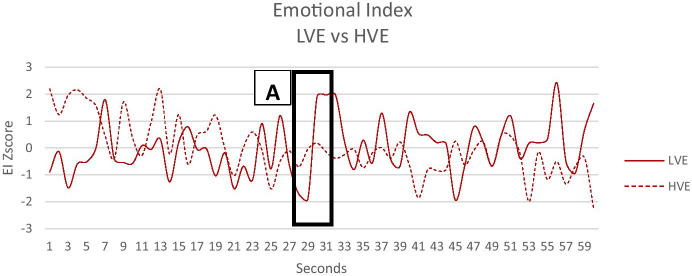
Emotional Index for LVE and HVE. Black box **A** represent the modified segment of the war images

**Fig. 4:**
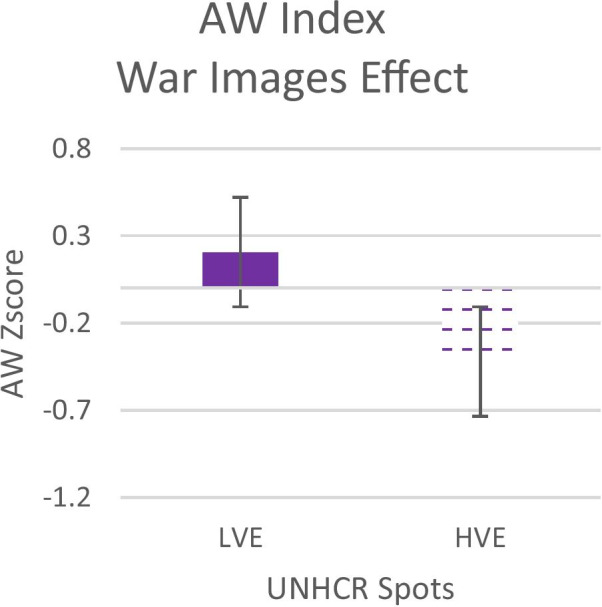
The graphs represent the AW Index for all participants during the spot after the war images' of LVE and HVE. Error bars represent standard error

Independent sample t-test analysis on AW index and Emotional index highlight significant differences between LVE and HVE after the visualization of victim images in the war. Instead, no significant difference before the modifications (secs 1–30) has been observed, as the spots were the same. The two neurophysiological indexes differed after the modifications: 1) for the Approach-Withdrawal index, lower values were found for the HVE than for the LVE (t = 2.44; p = 0.02) (see Fig. 3); 2) for the Emotional Index lower values were found for the HVE than for LVE (t = 2.34; p = 0.02) (See Fig. 4), confirming our H_2_.

### Call to action effect in cognitive response and visual attention

**Fig. 5:**
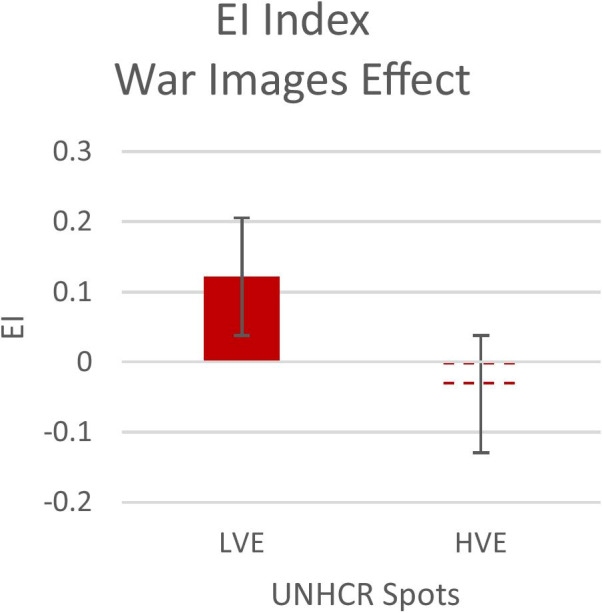
The graphs represent the Emotional Index for all participants during the spot after the war images' of LVE and HVE. Error bars represent standard error

**Fig. 6:**
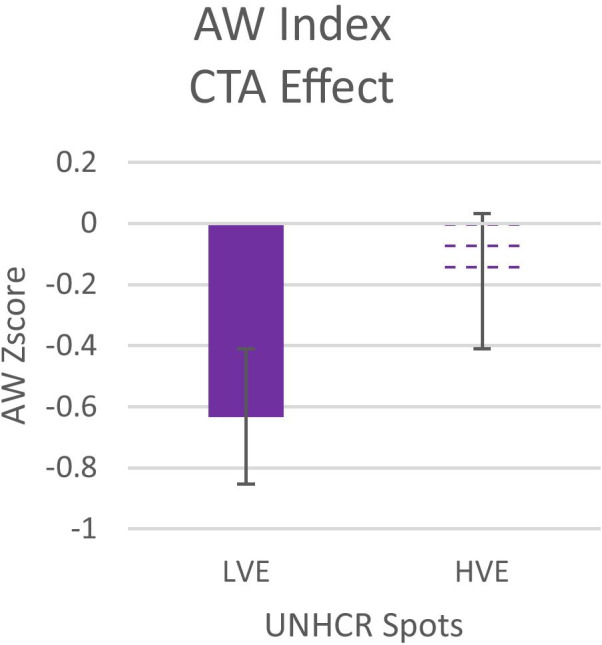
The graph represents the AW Index for all participants during the CTA scene of LVE and HVE. Error bars represent standard error

**Fig. 7:**
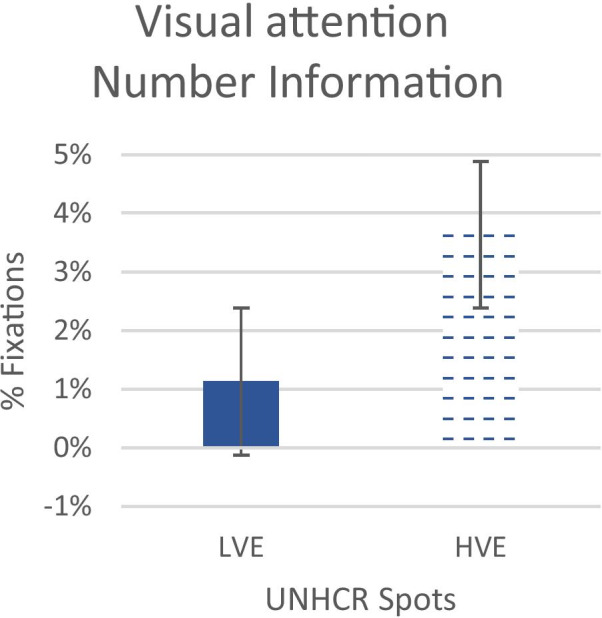
The graph represents the % Fixations for all participants during the final CTA in the number of information AOI for LVE and HVE. Error bars represent standard error

To evaluate the effect of the changes in the Call to Action on the Approach-Withdrawal index with respect to the whole spot (see Fig. 1 Box B), the Z Score of the index has been calculated for each second. In both spots, the values of AW index are negative in the CTA segment, but an independent sample t-test highlighted an increase in AW in the HVE spot compared to LVE (t = 2.10; p = 0.01) (see Fig. 5), showing that the CTA has a more positive effect in terms of approach on the spot in the HVE than in the LVE spot. Furthermore, an independent-sample t-test on fixation percentages on this segment showed that there is an increase in the fixation on the information number in the CTA for the HVE spot (independent sample t-test; t = -5,54; p < 0,0001) than in the LVE spot (See Fig. 6), confirming our H_3_ (Fig. 7).

These results support previous literature findings of the importance of creating empathy for a potential donor using proper emotions. As expected, our experiments also demonstrate that neuromarketing techniques are an appropriate methodology to measure consumer behaviour while watching advertising.

## Discussion and implications

Based on recent debates about non-profit advertising's effectiveness (Bergh & Reinstein, 2020; Kim, 2014) and the development of neuromarketing research in the last decade (Cherubino et al., 2019), we examined how the later can help to improve and predict the first one.

In this research with the UNHCR organization, an advertising campaign that had not received the expected impact (LVE) was analyzed, changed and successfully aired. Particularly, creativity weaknesses were identified, such as neutral and positive emotions and little emphasis on the CTA. After recognizing these problems, thanks to neuromarketing techniques, the images that could be modified were identified by neuromarketing analysis and considering the existing literature about non-profit advertising campaigns; a new spot (HVE) was created then released on TV. After a period of transmission, the UNHCR organization communicated a positive impact on real calls for donations. The number of calls asking for information about the testamentary legacy and people's subscription had increased considerably compared to the previous campaign, which confirms our H1. In the second recording session, the new spot (HVE) was also analyzed in a post-test study to identify whether and how the weaknesses identified in the first spot had been solved with the change in the creative strategy and its impact on the neurophysiological marker used in this research. This study's evidence implies recognizing a role for neuromarketing tools to calibrate the best creative strategy and potential to predict a non-profit advertising message framing's success. As a support to the original contribution of neuromarketing techniques, we also observe that results obtained in this study with the classical interview about the recalled memories and the level pleasantness failed to report any significative indications to solve the inefficacy of the version first of the spot.

### Theoretical implications

Results confirm theories about the type of emotions to evoke in non-profit advertising, and it gives indications regarding the need for generating a balance between positive and negative images as more suitable for the aim of the communication. Despite the fact that Allred and Amos (2018) confirm that too strong images of disgust showed lower donation intention, we found that images showing the reality of the war situation increased the calls for donations. Moreover, our technique shows a clear advantage over traditional marketing techniques to evaluate message framing and the subject’s perception.

### Managerial implications

The present findings have important implications for solving problems on advertising effectiveness. Results demonstrate that neuromarketing techniques are optimal to identify problems in real existing communications. Particularly, the neuromarketing approach has the potential to identify and modify material that does not appeal to consumers. Based on neurophysiological responses, the suggestions could shed light for practitioners on the strategy to use when the objective is an increase in the calls and subscriptions for donations. This is crucial for charity association such as UNHCR, where an increase in spot performance can have a real impact on people's lives.

## Conclusions and future research

The results obtained and described in this research highlight the advantages of more synergy between research and charity associations to frame a charity message: both could benefit from this integration. In particular, the present research contributes to the growing body of knowledge about the effectiveness of neuromarketing methods in improving the success of an ad, in general, and on how to create effective non-profit advertising, applying neuromarketing techniques as efficient tools to check coherence between copy strategy and elicited cognitive and emotional perception taking in account the more advanced theory to enhance non-profit communication. Further work should focus on different types of charity campaigns to identify a set of neurophysiological markers that can predict a communication's performance. Moreover, there is the need to identify population segments that can be more or less open/useful for targeting such communications, focusing on gender differences, age and socio-cultural stratification. Regarding theoretical implications, this will help increase the results in specific segment cases and identify if there are differences between them. Regarding managerial implications, it would help to target specific campaigns identified with particular segments and thus lower positioning costs. These topics are reserved for future work. Nevertheless, the study demonstrated the possibility and the interest to better investigate the cerebral and emotional reactions, together with visual attention as possible predictors of message framing efficacy of advertisements.

## Appendix

Some images that were changed for a better creativity strategy from LVE to HVE:

**Table:**

	LVE (First Test Session) https://www.youtube.com/watch?v=v1RQrovxb2g	HVE (Second Test Session) https://www.youtube.com/watch?v=aBGQSqjCpo0
Victims War Images substitution from sec.27; (Box A in Fig. 1 and Fig. 2)		
CTA without and with “progressive highlight effect” on the numbers in the AOI “call for information number”; (box B in Fig. 1)		

## Footnotes

1 Please click on https://www.youtube.com/watch?v=v1RQrovxb2g to watch the complete first version of the spot.

2 Please click on https://www.youtube.com/watch?v=aBGQSqjCpo0 to watch the complete second version of the spot.
